# Downregulation of Choline Kinase-Alpha Enhances Autophagy in Tamoxifen-Resistant Breast Cancer Cells

**DOI:** 10.1371/journal.pone.0141110

**Published:** 2015-10-23

**Authors:** Hoe Suk Kim, Lianji Tian, Minji Jung, Sul Ki Choi, Yujin Sun, Hyeonjin Kim, Woo Kyung Moon

**Affiliations:** 1 Department of Biomedical Science, College of Medicine, Seoul National University, 103 Daehak-ro, Jongno-gu, Seoul 110–799, Korea; 2 Department of Radiology, Seoul National University Hospital, 101 Daehak-ro, Jongno-gu, Seoul 110–744, Korea; Baylor College of Medicine, UNITED STATES

## Abstract

Choline kinase-α (Chk-α) and autophagy have gained much attention, as they relate to the drug-resistance of breast cancer. Here, we explored the potential connection between Chk-α and autophagy in the mechanisms driving to tamoxifen (TAM) resistance, in estrogen receptor positive (ER+) breast cancer cells (BCCs). Human BCC lines (MCF-7 and TAM-resistant MCF-7 (MCF-7/TAM) cells) were used. Chk-α expression and activity was suppressed by the transduction of shRNA (shChk-α) with lentivirus and treatment with CK37, a Chk-α inhibitor. MCF-7/TAM cells had higher Chk-α expression and phosphocholine levels than MCF-7 cells. A specific downregulation of Chk-α by the transduction of shChk-α exhibited a significant decrease in phosphocholine levels in MCF-7 and MCF-7/TAM cells. The autophagy-related protein, cleaved microtubule-associated protein light chain 3 (LC3) and autophagosome-like structures were significantly increased in shChk-α-transduced or CK37-treated MCF-7 and MCF-7/TAM cells. The downregulation of Chk-α attenuated the phosphorylation of AKT, ERK1/2, and mTOR in both MCF-7 and MCF-7/TAM cells. In MCF-7 cells, the downregulation of Chk-α resulted in an induction of autophagy, a decreased proliferation ability and an activation of caspase-3. In MCF-7/TAM cells, despite a significant decrease in proliferation ability and an increase in the percentage of cells in the G0/G1 phase of the cell cycle, the downregulation of Chk-α did not induced caspase-dependent cell death and further enhanced autophagy and G0/G1 phase arrest. An autophagy inhibitor, methyladenine (3-MA) induced death and attenuated the level of elevated LC3 in MCF-7/TAM cells. Elucidating the interplay between choline metabolism and autophagy will provide unique opportunities to identify new therapeutic targets and develop novel treatment strategies that preferentially target TAM-resistance.

## Introduction

Tamoxifen (TAM), an antagonist of the estrogen receptor, is the most commonly used treatment for patients with estrogen receptor positive (ER+) breast cancer [1]. However, approximately 30% of ER+ breast cancers do not respond to TAM treatment, and the majority of tumors that initially respond to TAM treatment develop resistance over time [2]. These resistant cells survive in a dormant state and hide for years or decades, ultimately giving rise to incurable metastases [3,4]. Therefore, understanding the mechanism of TAM-resistance is important in the treatment of recurrent and metastatic ER+ breast cancer.

Choline-containing metabolites are non-invasive metabolic biomarkers used to identify malignant transformation and to determine the therapeutic response of cancer cells *in vitro* and *in vivo* using magnetic resonance spectroscopy [5,6]. A high level of phosphocholine (PC) induced by the increased expression or activity of choline kinase-α (Chk-α) is one of the metabolic characteristics of diverse types of human cancers [7–11]. Chk-α has been proposed as a prognostic marker for cancer progression and therapeutic resistance as well as a molecular target for the development of novel anti-cancer drugs [12]. The Chk-α overexpression induces the invasiveness and drug resistance in breast cancer cells [13,14]. Conversely, the Chk-α downregulation with small interfering RNA inhibits cell proliferation and markedly decreases anchorage-independent survival in malignant cancer cells through simultaneous attenuation of phosphatidylinositol 3-kinase (PI3K)/AKT and mitogen-activated protein kinases (MAPK) signaling [15].

Autophagy is a major catabolic pathway for the delivery of damaged or superfluous proteins to lysosomes or the vacuole and subsequent degradation by the cell's own lysosomal system [16]. Autophagy, which plays a dual role in both cell survival and cell death, limits tumor necrosis and inflammation and mitigates genome damage in cancer cells in response to metabolic stress, thereby protecting the cancer cell [17–19]. A recent studies have reported a critical role of autophagy in regulating treatment resistance and tumor dormancy related with eventual tumor regrowth and progression [20]. Many cancer therapeutic agents have been reported to induce autophagy and there is growing evidence for autophagy induction as a key drug resistance mechanism within cancer treatment [4,21]. TAM-resistant cells show an ability to undergo anti-estrogen-induced autophagy without the induction of caspase-dependent cell death, suggesting that autophagy as a key regulator of the anti-estrogen resistance in ER+ BCCs [22,23]. In addition to driving drug resistance, autophagic pathways share some signaling molecules, including PI3K/AKT and mammalian target of rapamycin (mTOR), which regulate cell growth and protein synthesis in response to nutrient and growth factor availability [24]. The MAPK signaling pathway is involved in both the induction of autophagy and the maturation of the autophagosome [25].

The aforementioned studies indicate that Chk-α and autophagy might be critical components of the process that leads to therapeutic resistance, dormancy and delayed recurrence of breast cancer. Nevertheless, the relationship between Chk-α and autophagy in TAM-resistant BCCs is not well understood. To better understand the relationship between Chk-α and autophagy, we here investigated the autophagy-related proteins as well as autophagy-controlled signaling pathways (PI3K/AKT, mTOR and MAPK) in Chk-α-dwonregulated ER+ BCCs (MCF-7) and TAM-resistant BCCs (MCF-7/TAM) by the transduction of small hairpin RNA or short hairpin RNA (shRNA) and treatment with the Chk-α inhibitor, CK37. MCF-7 cells exhibited both the induction of autophagy and caspase-dependent apoptosis. In contrast, the downregulation of Chk-α enhanced the autophagy and cell dormancy of MCF-7/TAM cells that have acquired TAM-resistance. Our studies suggest that the alteration of Chk-α expression and activity in BCCs might modulate the autophagy and the regulation of both Chk-α and autophagy in cancer cells could abrogate tumor resistance to TAM.

## Materials and Methods

### Cell culture

The ER+ human BCC line, MCF-7, was obtained from the ATCC (Manassas, VA, USA). and routinely cultured in Dulbecco's Modified Eagle's Media (DMEM) containing 10% fetal bovine serum (Invitrogen corp., Carlsbad, CA, USA), 100 units/mL penicillin, and 100 μg/mL streptomycin (Sigma-Aldrich, St. Louis, MO, USA). TAM-resistant cell line, MCF-7/TAM, derived from MCF-7 cell was obtained from Dr. Sang-kyu Ye (Department of pharmacology, Seoul national university college of medicine). MCF-7/TAM were continuously cultured in the medium as described above containing additional 3 μmol/L TAM (Sigma-Aldrich) for at least six months, along with the parental MCF-7-cells under identical culture conditions except that the control cells were treated with 0.1% ethanol. The two cell lines were grown side by side at all times. Cell cultures were maintained at 37°C in a humidified atmosphere of 95% air/5% CO_2_.

### Establishment of Chk-α downregulated cells using lentivirus containing the Chk-α shRNA

The lentiviral-derived plasmids that carried the Chk-α shRNA (shChk-α) and green fluorescent protein (GFP) were provided by Thermo Fisher Scientific Inc. (no. RHS4531). 293T cells were transfected with lentiviral packing plasmids and shChk-α/GFP plasmids using Lipofectamine 2000 (Invitrogen) for lentivirus packaging, and 48 h later, the virus-containing supernatant medium was collected, filtered, and concentrated by ultracentrifugation. In brief, 1x10^5^ BCCs were seeded in a six-well plate and infected with lentivirus containing the shChk-α/GFP. After 6 h, the medium was removed and replaced with fresh medium. The cells were incubated for 72 h, and GFP expressing cells were sorted using a FACSCalibur flow cytometer (BD Biosciences, Franklin Lakes, NJ, USA) equipped with a 530-nm filter (bandwidth, ± 15 nm), a 585-nm filter (bandwidth, ± 21 nm), and analyzed using a CellQuest software (BD Biosciences). The downregulation of Chk-α in transduced cells was evaluated by RT-PCR and Western blot.

### Real-time RT-PCR analysis

After cell sorting, MCF-7 and MCF-7/TAM cells were seeded in six-well plates in DMEM culture medium and incubated in a humidified CO_2_ incubator (5% CO_2_, 37°C). To investigate the expression of Chk-α and Chk-β, RT-PCR was performed. Total RNA was extracted from cultured cells using TRIzol Reagent (Invitrogen). RNA quantity and quality were determined using a NanoDrop spectrophotometer (Thermo Fisher Scientific Inc. Waltham, MA USA). cDNA was produced by using SuperScript II reverse transcriptase (Invitrogen). The following primers were used: Chk-α, 5′-CTTGGTGATGAGCCTCGGAA-3′, and 5′-AAGTGACCTCTCTGCGAGAA-3′; Chk-β, 5′-AGTCTCGGTTCCAGTTCTAC-3′, and 5′-CTTCTGCTCGTTGTTCCTCC-3′; and β-actin, 5′-CCAACCGCGAGAAGATGACC-3′ and 5′-GGAGTCCATCACGATGCCAG-3′. Real-time PCR reactions were run on the ABI PRISM^®^ 7900 using a SYBR Green PCR master mix (Applied Biosystems, Foster City, CA, USA). The results were analyzed by the ΔCt method, which reflects the difference in threshold for the target gene relative to that of β-actin in each sample.

### Western blot analysis

The cells were lysed in RIPA buffer containing a protease inhibitor cocktail (Sigma-Aldrich), and the proteins were resolved by sodium dodecyl sulfate polyacrylamide gel electrophoresis (SDS-PAGE) for 4 h at room temperature and transferred to nitrocellulose membranes for 2 h at 4°C. The membranes were blocked with 5% skim milk in Tris-buffered saline and incubated with primary antibodies overnight at 4°C followed by an incubation with a horseradish peroxidase-conjugated secondary antibody (Santa Cruz Biotechnology, Santa Cruz, CA, USA) at room temperature for 30 min. The blots were developed using Enhanced Chemiluminescence Reagents (Amersham Biosciences, Piscataway, NJ, USA). The relative intensity of the bands observed by Western blotting was analyzed using Image J software. The following antibodies were used in this study: Anti-phospho AKT (Cell Signaling Technology, Danvers, MA, USA), anti-AKT (Cell Signaling Technology), anti-phospho ERK1/2 (Cell Signaling Technology), anti-ERK1/2 (Cell Signaling Technology), anti-phospho-mTOR (Cell Signaling Technology), anti-mTOR (Cell Signaling Technology), anti- microtubule-associated protein light chain 3 (LC3) (Cell Signaling Technology), anti-beclin-1 (Cell Signaling Technology), anti-p62 (Cell Signaling Technology), anti-p21 (Cell Signaling Technology), anti-p27 (Cell Signaling Technology) anti-Chk-α (Proteintech Group, Inc., Chicago, IL, USA) and anti-β-actin (Sigma-Aldrich) antibodies. All antibodies were used according to the manufacturers’ instructions.

### Cell Proliferation, cell cycle and cell viability assays

*In vitro* cell proliferation was assessed using the 3-(4, 5-dimethylthiazol-2-yl)-2,5-diphenyl tetrazolium bromide (MTT) assay. Briefly, 1 × 10^3^ cells were allowed to adhere for 24 h under a high humid environment in 5% CO_2_ at 37°C in 96-well culture plates. After 24–72 h, MTT solution (a final concentration of 1 mg·mL^−1^) was added, and the cells were incubated for 2 h. At the end of the incubation period, the reaction mixture was carefully removed, and 200 μl of dimethyl sulfoxide was added to each well. The plates were kept on a rocker shaker for 10 min at 26°C and then read at 540 nm using a Multi-detection Microplate Reader (Bio-Tek instruments, Winooski, VT, USA).

After inhibitor of autophagy, *in vitro* cell viability was evaluated by MTT assay. Briefly, 1 × 10^4^ cells were allowed to adhere for 24 h in 96-well culture plates. Cells were incubated in medium supplemented with 3-Methyladenine (3-MA, Sigma-Aldrich) which is widely used as an autophagy inhibitor. After treatment with 3-MA (5 mM) for 24–72 h, cell viability was measured by the MTT method as described above.

Cell cycle assays were performed to determine whether shChk-α transduction regulates the growth phase and death of BCCs. Cells were trypsinized and centrifuged at 300 × *g* (1000 rpm) for 5 min, then resuspended and fixed with 70% ice-cold ethanol for 60 min. The fixed cells were centrifuged, washed and resuspended in phosphate-buffered saline (PBS) containing DNase-free RNase. After a 30-min incubation, propidium iodide (PI, 50 μg/ml) was added to the resuspended cells and incubated for an additional 15 min in the dark. PI fluorescence was analyzed using flow cytometry (BD Biosciences). Cell subpopulations in G0/G1, S and G2/M phases and apoptosis were calculated by gating analysis based on differences in DNA content. At least 20000 cells were analyzed per sample. Cell proliferation characters were indexed by the ratio of cells in S-phase.

### Immunocytochemistry

For immunocytochemical stains, cells were cultured on eight-well chamber slides, rinsed in PBS and fixed with 2% paraformaldehyde for 30 min at 4°C. The fixed cells were blocked with 2% bovine serum albumin (Sigma-Aldrich) in PBS, reacted with anti-LC3, anti-Ki67 (Santa Cruz Biotechnology) and anti-cleaved caspase-3 (Sigma-Aldrich) antibodies, visualized using secondary antibodies conjugated to Alexa 594 (Invitrogen) and counterstained with 4',6-diamidino-2-phenylindole (DAPI). These fluorescently stained cells were then observed under a fluorescence microscope as previously described using LAS software (Leica) for image acquisition.

### 1H-nuclear magnetic resonance (1H- NMR) spectroscopy analysis

The cells were harvested, collected as cell pellets containing 3x10^7^ cells per sample, and stored at -80°C until ready for 1H-NMR spectroscopy analysis. 1H-NMR spectroscopy was performed using methods published previously by Blankenberg *et*. *al*. [26]. Frozen cell pellets were thawed with D_2_O made in PBS, with 1.5 mM sodium 3-(trimethylsilyl)-propionate-2,2,3,3-d4 (TSP; Cambridge Isotope Laboratories, Inc., Andover, MA, USA) added as an internal standard, suspended in a final volume of 900 μL, centrifuged to remove precipitates and then placed immediately on ice until data acquisition. One-dimensional standard 1H-NMR and CPMG spectra were acquired on a Bruker Avance 600 system (14.1 T) equipped with a 5-mm TXI cryoprobe spectrometer (Bruker BioSpin Corp., Billerica,MA, USA). Spectra were acquired at 20°C±1°C with the following sequence parameters; flip angle = 90°, spectral width = 16 kHz, relaxation delay = 2 s, 32 k data points, and 128 scans. Chemical shifts were calibrated relative to the TSP signal at 0 ppm. 1H-NMR spectra were processed using MestReNova software (Mestrelab research) and MRUI software [27]. The time-domain data were apodized with an exponential function (1 Hz) and then Fourier-transformed followed by phase- and baseline-correction. The spectra were referenced to the TSP signal at 0.00 ppm. The assigned resonances were also referenced to the alanine signal at 1.48 ppm in cases where the TSP signal was split due to protein binding. To reduce the complexity of the 1H- NMR spectroscopy data for the subsequent multivariate analysis, the spectra were binned by 0.005-ppm intervals and normalized by integration values over the region of 0.99–5.59 ppm. The above binning interval was chosen to quantify choline (Cho), phosphocholine (PC) and glycerophosphocholine (GPC) more accurately by allowing each of the signals to span at least two bins. The binning and normalizations were performed using an in-house Perl program.

### Data analysis

All data were presented as the mean ± standard deviation in five independent experiments and were analyzed using one way ANOVA followed by a T-test. Mean differences with p≤0.05 were considered statistically significant.

## Results

### TAM-resistant cells exhibited an increase in Chk-α expression and intracellular PC levels

**Fig 1:**
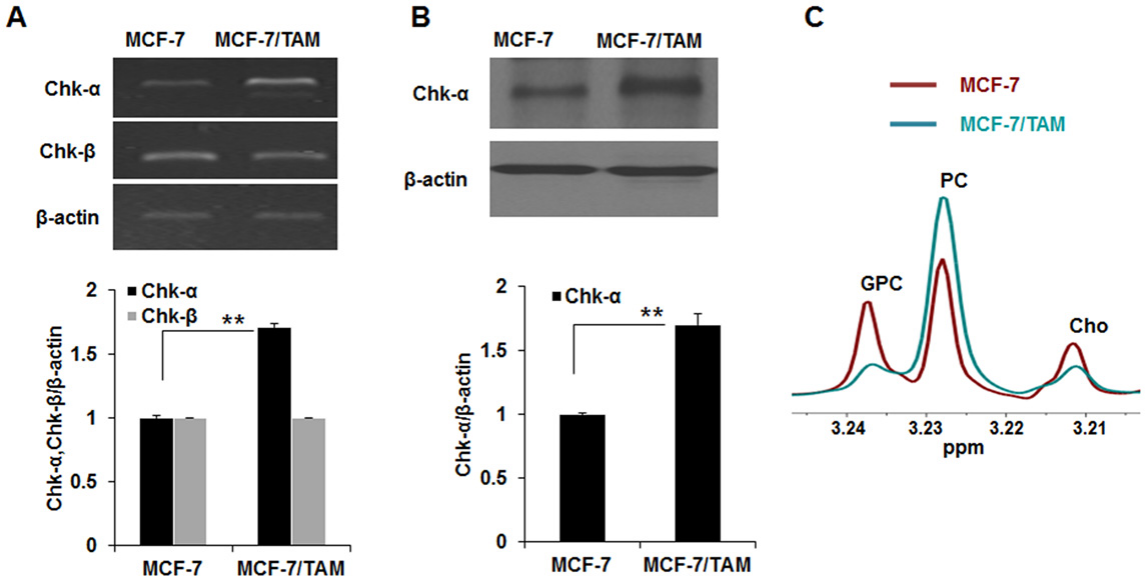
Choline kinase-α (Chk-α) expression and choline-containing metabolites increased in tamoxifen-resistant breast cancer cells, MCF-7/TAM. (A) RT-PCR analysis of Chk-α and β in MCF-7 and MCF-7/TAM cells. The level of Chk-α mRNA was higher in MCF-7/TAM cells and there was no different in the level of Chk-β mRNA between MCF-7 cells and MCF-7/TAM cells. (B) Western blot analysis of Chk-α in MCF-7 and MCF-7/TAM cells. Chk-α expression was significantly increased in MCF-7/TAM cells compared to MCF-7 cells. (C) A representative 1H-NMR spectra of choline-containing metabolites (choline (Cho), phosphocholine (PC) and glycerophosphocholine (GPC)) of MCF-7 and MCF-7/TAM cells. Higher PC level was observed in MCF-7/TAM cells relative to MCF-7 cells. Data are presented as the mean ± standard deviation of 3 independent experiments. ** *p*<0.001.

Chk-α and β expression in MCF-7 and MCF-7/TAM cells was analyzed to determine whether Chk-α expression was altered in TAM-resistant BCCs. Real-time RT-PCR analysis showed that the expression level of Chk-α mRNA was increased in MCF-7 cells treated with TAM (1–10 μM) (S1A Fig). Chk-α mRNA and protein, as analyzed by RT-PCR and Western blotting, increased up to 1.7-fold in MCF-7/TAM-resistant cells compared to MCF-7 cells, while Chk-β expression was no different between the MCF-7 and MCF-7/TAM cells (Fig 1A and 1B. *p<0.05.). Choline-containing metabolites in MCF-7 and MCF-7/TAM cells were analyzed using 1H-NMR spectroscopy. As shown in Fig 1C, PC was increased in MCF-7/TAM cells compared to MCF-7 cells, while GPC and Cho decreased. This result implies that the high level of Chk-α expression might be associated with the acquisition of TAM resistance in MCF-7 cells.

### The downregulation of Chk-α expression caused a significant reduction of PC levels

**Fig 2:**
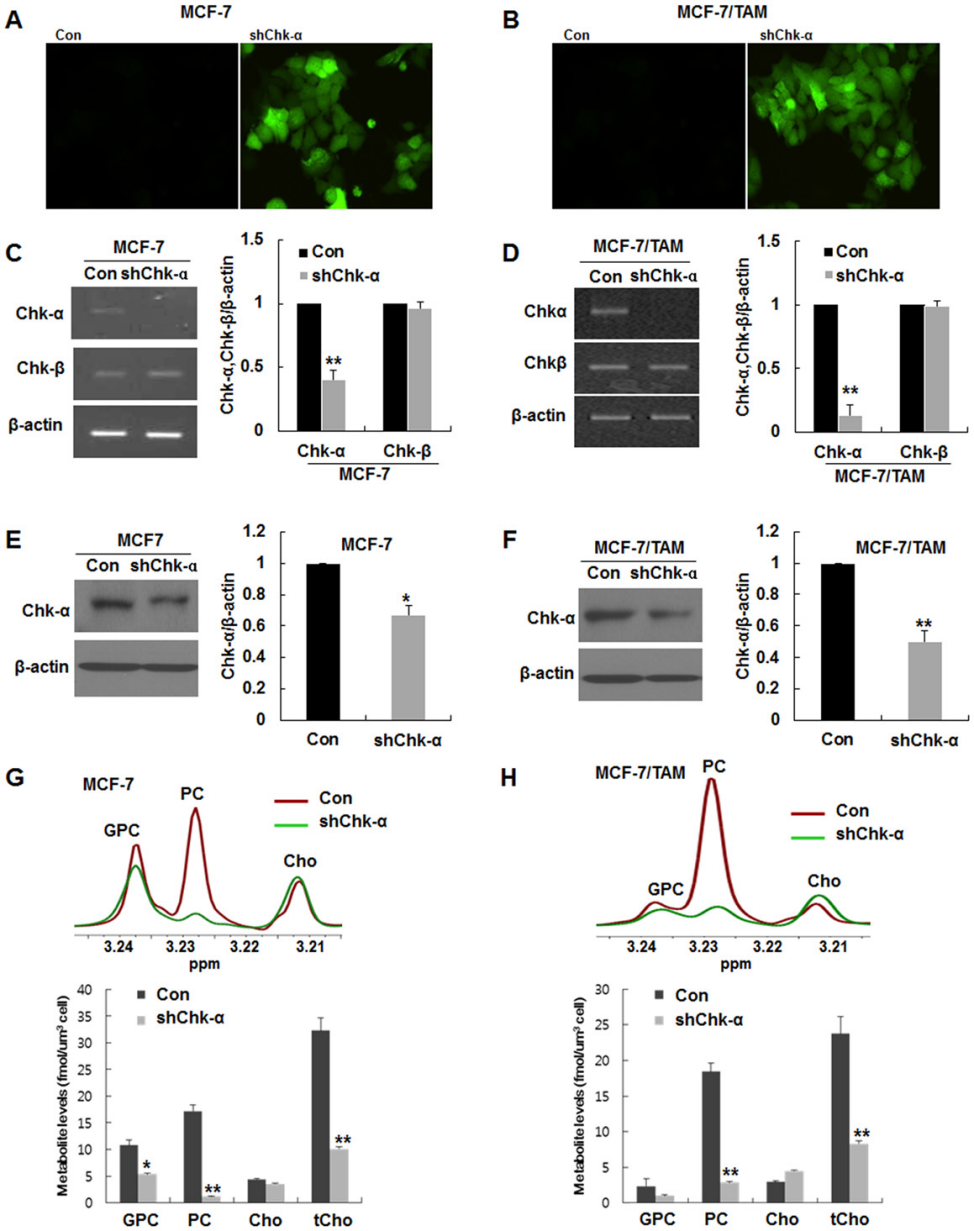
Establishment of choline kinase-α (Chk-α)-downregulated breast cancer cells and analysis of choline-containing metabolites. (A and B) Images of MCF-7-shChk-α and MCF-7/TAM-shChk-α cells transduced with lentivirus containing Chk-α shRNA and GFP. (C and D) RT-PCR analysis of Chk-α and β. A selective and significant decrease in Chk-α mRNA levels was detected in MCF-7-shChk-α and MCF-7/TAM-shChk-α cells. (E and F) Western blot analysis of Chk-α. Chk-α was significantly downregulated in MCF-7-shChk-α and MCF-7/TAM-shChk-α. (G and H) Analysis of 1H-NMR spectra of choline-containing metabolites, choline (Cho), phosphocholine (PC) and glycerophosphocholine (GPC). PC level remarkably decreased in MCF-7-shChk-α and MCF-7/TAM-shChk-α as compared to MCF-7 and MCF-7/TAM cells. Data are presented as the mean ± standard deviation of 5 independent experiments. * *p*<0.05 and ** *p*<0.001. shChk-α transduced vs control.

Next, Chk-α-downregulated BCCs (MCF-7-shChk-α and MCF-7/TAM-shChk-α) were established using lentivirus containing Chk-α shRNA and GFP (Fig 2A and 2B). As shown in Fig 2C and 2D, Chk-α mRNA levels were significantly decreased in MCF-7-shChk-α and MCF-7/TAM-shChk-α cells compared to MCF-7 and MCF-7/TAM cells (**p<0.001). However, the level of Chk-β mRNA was not changed in the MCF-7-shChk-α and MCF-7/TAM-shChk-α cells. Chk-α protein levels were significantly decreased in the MCF-7-shChk-α and MCF-7/TAM-shChk-α cells compared with the MCF-7 and MCF-7/TAM cells (Fig 2E and 2F, *p<0.05, **p<0.001). To analyze the alteration of choline-containing metabolites in shChk-α transduced cells, metabolite profiles were investigated using 1H- NMR spectroscopy. As we expected, PC levels of MCF-7-shChk-α and MCF-7/TAM-shChk-α cells remarkably decreased and were significantly lower than those of the MCF-7 and MCF-7/TAM cells (Fig 2G and 2H, **p<0.001). This result represent that Chk-α is a key role in maintaining high PC level in breast cancer cells.

### Transduction of Chk-α shRNA and treatment of Chk-α inhibitor, CK37 enhanced the expression of the autophagosomal marker, LC3 and p62 and the formation of autophagosomes in MCF-7/TAM cells

**Fig 3:**
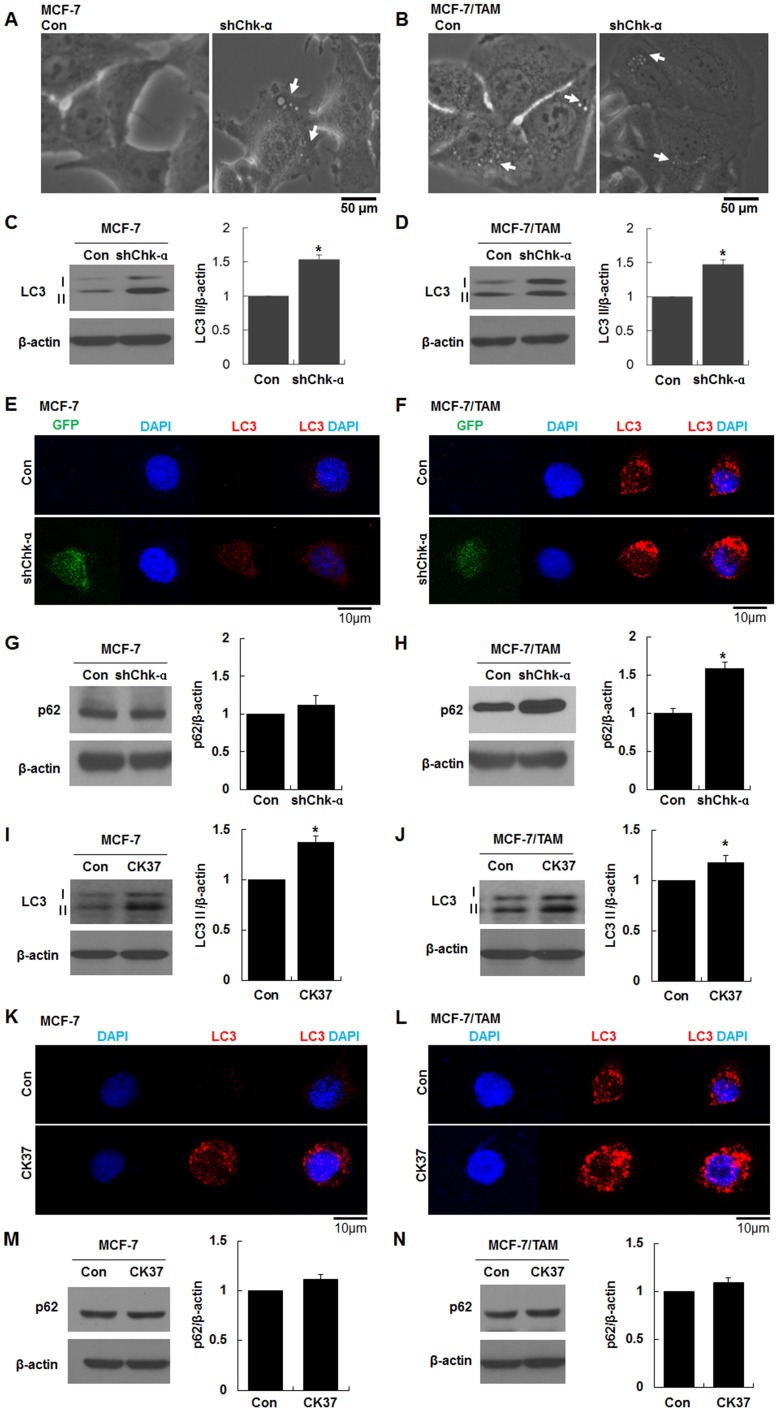
Downregulation of choline kinase-α (Chk-α) expression and activity enhanced the expression of the autophagosome marker, LC3 and P62 and the formation of autophagosomes in MCF-7/TAM cells. (A and B) Images of autophagic structure in MCF-7-shChk-α and MCF-7/TAM-shChk-α cells. Chk-α downregulation induced autophagic structures (arrow) in MCF-7 cells as well as MCF-7/TAM cells. (C and D) Western blot analysis of LC3. MCF-7-shChk-α and MCF-7/TAM-shChk-α cells exhibited a significantly higher LC3 as compared to MCF-7 and MCF-7/TAM cells. (E and F) Immunofluorescence images for LC3. Chk-α downregulation led to an increase in the number of autophagosomes stained with LC3 antibody. (G and H) Western blot analysis of p62. MCF-7/TAM-shChk-α cells exhibited a significantly higher p62 as compared to MCF-7/TAM cells. (I and J) Western blot analysis of LC3. Inhibition of Chk-α activity by treatment with CK37 significantly increased LC3 expression. (K and L) Immunofluorescence images for LC3. CK37 treatment resulted in an increase in the number of autophagosomes stained with LC3 antibody. (M and N) Western blot analysis of p62. CK37 treatment did not change the level of p62 as compared to untreated cells. Data are presented as the mean ± standard deviation of 5 independent experiments. *p<0.05. shChk-α transduced vs control and CK37-treated vs control.

TAM treatment increased LC3II levels in a dose-dependent manner in MCF-7 cells (S1B Fig). Interestingly, shChk-α transduction induced the formation of autophagosome-like structures in MCF-7 cells (Fig 3A). The autophagosome-like structures in MCF-7/TAM cells was further increased by shChk-α transduction (Fig 3B). About 1.5-fold increase in LC3II expression was observed in MCF-7-shChk-α and MCF-7/TAM-shChk-α cells compared to the MCF-7 and MCF-7/TAM cells (Fig 3C and 3D, *p<0.05), whereas beclin-1 levels were not altered (S2 Fig). Immunostaining of LC3, the autophagosomal marker showed that a large number of small clusters and intensely stained granules were mainly distributed in the cytoplasm near the nuclei of MCF-7/TAM, MCF-7-shChk-α and MCF-7/TAM-shChk-α cells (Fig 3E and 3F). MCF-7/TAM-shChk-α cells exhibited a strong increase in staining of LC3 compared to MCF-7/TAM cells (Fig 3F). Additional autophagy marker, p62 was analyzed in in MCF-7-shChk-α and MCF-7/TAM-shChk-α cells. MCF-7-shChk-α cell did not led to a significant increase in p62 compared to MCF-7 cells (Fig 3G). However, there is about 1.6-fold significant increase in p62 in MCF-7/TAM-shChk-α cells relative to MCF-7/TAM cells (*p<0.05, Fig 3H). Furthermore, we investigated whether the inhibition of Chk-α activity leads to autophagy. MCF-7 and MCF-7/TAM cells were incubated with 10 μM CK37, a Chk-α inhibitor for 24 h. The inhibition of Chk-α by CK37 induced a significant increase in LC3II expression in MCF-7 and MCF-7/TAM cells (Fig 3G and 3H, *p<0.05). As we expected, autophagosome formation detected by immunostaining of LC3 was increased in MCF-7 and MCF-7/TAM cells treated with CK37 (Fig 3I and 3J). CK37 treatment led to an increase in the size and number of autophagosome formation in MCF-7/TAM cells (Fig 3J). CK37 treatment caused MCF-7 and MCF-7/TAM cells to a slight increase in p62 (Fig 3M and 3N). These results demonstrated that the alteration of Chk-α expression or activity could be involved in regulating autophagy in MCF-7 and MCF-7/TAM cells.

### The downregulation of Chk-α decreased the phosphorylation levels of the ERK1/2, AKT and mTOR

**Fig 4:**
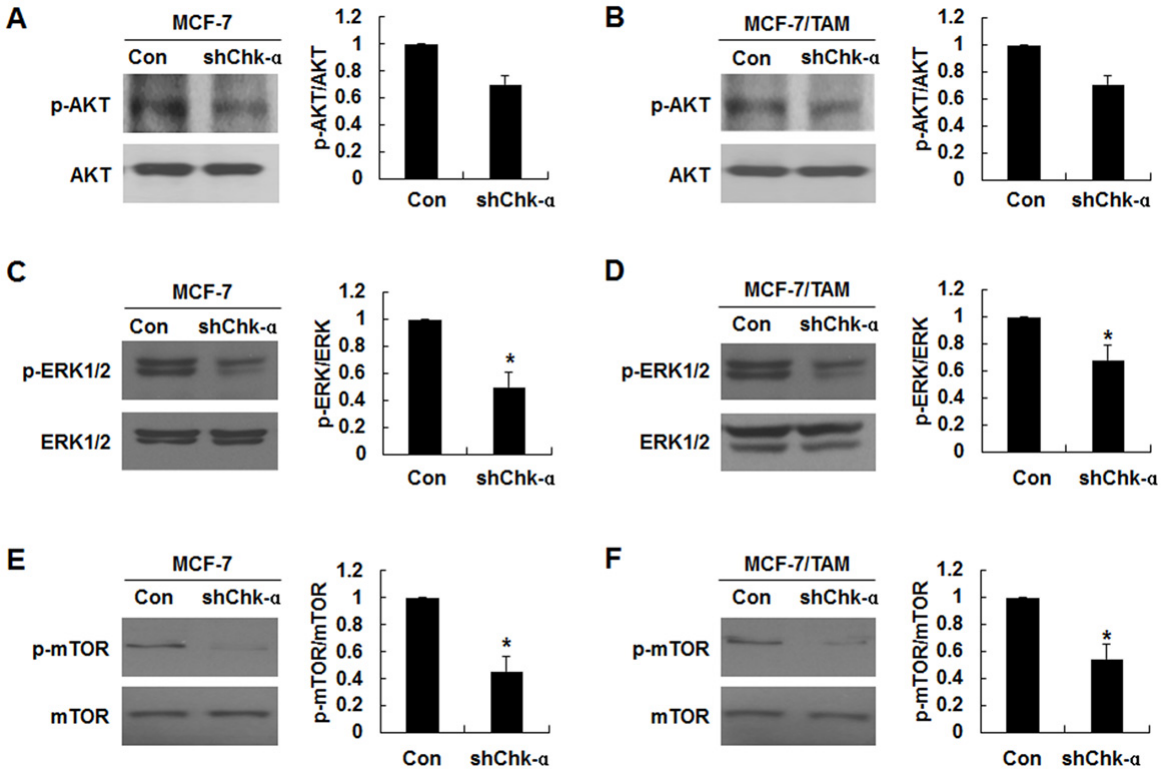
The downregulation of Chk-α attenuated the phosphorylation levels of ERK1/2, AKT and mTOR. (A) Western blot analysis of the phosphorylation of AKT. (B) Western blot analysis of the phosphorylation of ERK1/2 (C) Western blot analysis of the phosphorylation of mTOR. The phoshorylation levels of AKT, ERK1/2 and mTOR decreased MCF-7-shChk-α and MCF-7/TAM-shChk-α cells relative to MCF-7 and MCF-7/TAM cells. Data are presented as the mean ± standard deviation of 5 independent experiments. *p<0.05. shChk-α transduced vs control.

We investigated whether the MAPK, AKT, mTOR signaling pathways are associated with the autophagy induced by Chk-α downregulation. shChk-α transduction resulted in a slight decrease in the phosphorylation level of AKT at Ser473, but the difference in AKT phosphorylation levels between MCF-7-shChk-α and MCF-7/TAM-shChk-α cells and MCF-7 and MCF-7/TAM cells was not significant (Fig 4A and 4B). The significant attenuation of ERK1 and ERK2 phosphorylation at Thr202/Tyr204 and Thr185/Tyr187, respectively was observed in MCF-7-shChk-α and MCF-7/TAM-shChk-α cells compared with MCF-7 and MCF-7/TAM cells (Fig 4C and 4D, *p<0.05). Moreover, a significant decrease in the phosphorylation of mTOR at Ser 2448 was also observed in MCF-7-shChk-α and MCF-7/TAM-shChk-α cells as compared to MCF-7 and MCF-7/TAM cells (Fig 4E and 4F, *p<0.05). These results together suggest that an attenuated phosphorylation of ERK1/2, AKT or mTOR might be involved in autophagy induction in MCF-7-shChk-α and MCF-7/TAM-shChk-α cells.

### The downregulation of Chk-α controlled the cell growth and cell cycle in MCF-7/TAM cells but induced caspase-dependent death in MCF-7 cells

**Fig 5:**
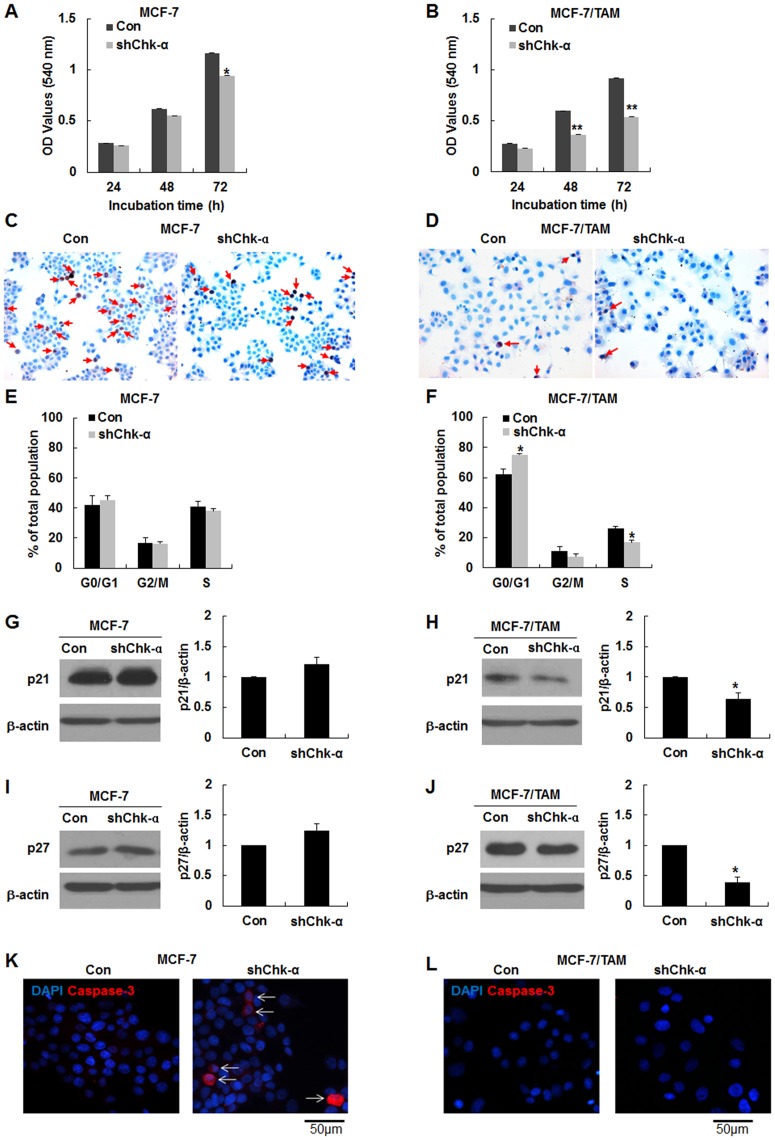
The downregulation of Chk-α attenuated the cell growth rate and enhanced G0/G1 fraction and in MCF-7/TAM but induced caspase-dependent death in MCF-7 cells. (A and B) MTT assay in assessing cell viability and growth. MCF-7-shChk-α and MCF-7/TAM-shChk-α cells exhibited the decreased cell viability and growth ability compared to MCF-7 and MCF-7/TAM cells. (C and D) Immunostaing of Ki67. The downregulation of Chk-α led to a decrease in the number of Ki-67 positive cells (red arrow). (E and F) Cell cycle analysis using propidium iodide staining and flow cytometry. The significant increase and decrease in the population in G0/G1 and the S proportion in MCF-7/TAM-shChk-α cells as compared to MCF-7/TAM cells. (G and H) Western blot analysis of the p21. p21 level was higher in MCF-7 and MCF-7-shChk-α cells relative to MCF-7/TAM and MCF-7/TAM-shChk-α cells. The downregulation of Chk-α caused to a significant decrease in p21 level in MCF-7/TAM cells. (I and J) Western blot analysis of the p27. MCF-7/TAM and MCF-7/TAM-shChk-α cells expressed p27 highly as compared to MCF-7 and MCF-7-shChk-α cells. The downregulation of Chk-α caused to a significant decrease in p27 level in MCF-7/TAM cells. (K and L) Immunofluorescence image of cleaved caspase-3. Activated caspase-3 (white arrow) was observed in MCF-7-shChk-α cells but not inMCF-7/TAM-shChk-α cells. Data are presented as the mean ± standard deviation of 5 independent experiments. **p<0.001, *p<0.05. shChk-α transduced vs control.

As shown in MTT assay, the growth of MCF-7-shChk-α cell decreased at 72 h when compared with those of MCF-7 cell (Fig 5A, *p<0.05). MCF-7/TAM cell was a slower growing cell line compared to the MCF-7 cell. Moreover, the Chk-α downregulation resulted in a significant decrease in the proliferation and viability of MCF-7/TAM cell at 48 and 72 h (Fig 5B, **p<0.001). The monoclonal antibody Ki-67 was tested as a marker of proliferating cells. Many populations of MCF-7 cells were positive for Ki-67 and MCF-7-shChk-α displayed the decreased Ki-67 positive population (Fig 5C). MCF-7/TAM and MCF-7/TAM-shChk-α cells exhibited a lower Ki-67 positive population than parental MCF-7 cells (Fig 5D). Next, we investigated that autophagy induced by Chk-α downregulation plays a role in controlling cell cycle, cell proliferation, cell viability or cell death. Cell cycle analysis assessed by flow cytometry showed that the cell proportion in G0/G1, G2/M and S cell cycle phase of MCF-7 cells were 42.0 ± 6.5%, 16.8 ± 3.2% and 41.2 ± 3.1%, respectively and the cell proportion in G0/G1, G2/M and S cell cycle phase of MCF-7-shChk-α cells were 45.5 ± 3.0%, 16.3 ± 1.5% and 38.2 ± 1.6%, respectively (Fig 5E). There was no alteration of cell cycle between MCF-7 cells and MCF-7-shChk-α cells. In MCF-7/TAM cells, 62.3 ± 3.2%, 11.5 ± 2.5% and 26.2 ± 1.5% of the gated population were in G0/G1, G2/M and S cell cycle phase, respectively and the cell proportion in G0/G1, G2/M and S cell cycle phase of MCF-7/TAM-shChk-α cells were 75.2 ± 0.8%, 7.7 ± 1.4% and 17.1 ± 1.2% (Fig 5F). MCF-7/TAM as well as MCF-7/TAM-shChk-α exhibited a significantly higher G0/G1 fraction compare to parental MCF-7(*p<0.05). Next we investigated the levels of p21 and p27, cyclin-dependent kinase inhibitors, which are related to the G0/G1 arrest and resulted in anti-proliferative effect. p21 decreased remarkably in MCF-7/TAM and MCF-7/TAM-shChk-α cells relative to MCF-7 and MCF-7-shChk-α cells (Fig 5G and 5H). p27 was greatly elevated in MCF-7/TAM and MCF-7/TAM-shChk-α cells relative to MCF-7 and MCF-7-shChk-α cells (Fig 5I and 5J). Unexpectedly, p21 and p27 levels were found to be significantly decreased in MCF-7/TAM-shChk-α cells compared to MCF-7/TAM cells (*p<0.05). We further examined additional reason for the decreased cell growth and viability in MCF-7-shChk-α and MCF-7/TAM-shChk-α cells. Interestingly, activated caspase-3 was observed in MCF-7-shChk-α cells but not inMCF-7/TAM-shChk-α cells (Fig 5K and 5L). These findings provided that a decreased cell viability in MCF-7-shChk-α is likely due to the apoptotic death, whereas a decrease in cell growth in MCF-7/TAM-shChk-α may be associated with an increase in the proportion of G0/G1 phase as well as an attenuation of cell growth rate to confer cell dormancy.

### Enhanced autophagy is being in MCF-7/TAM and MCF-7/TAM-shChk-α cells pays a role in protecting the cells from TAM

**Fig 6:**
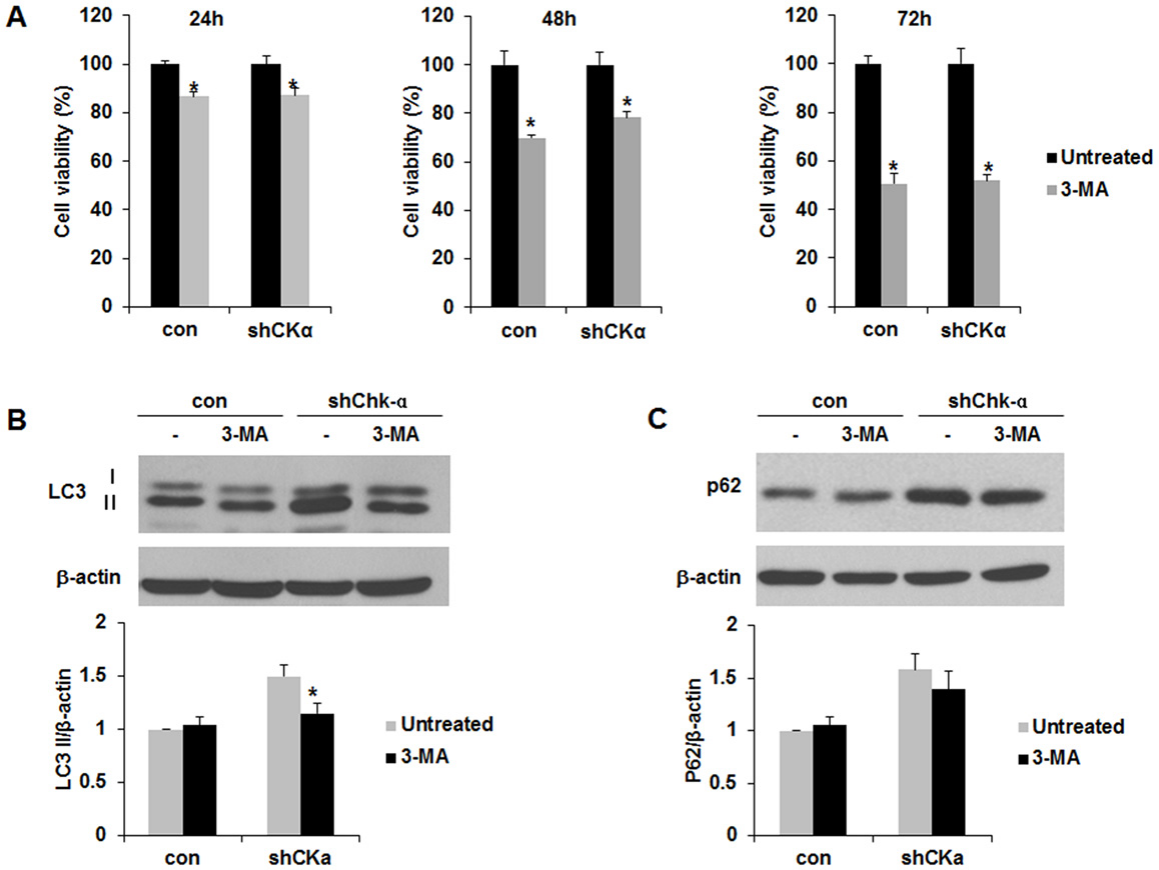
The autophagy enhanced by downregulation of Chk-α contributed to protect MCF-7/TAM cells from tamoxifen. (A) MTT assay for cell viability analysis. Treatment with 3-methyladenine (3-MA), an autophagy inhibitor induced the cell death in MCF-7/TAM and MCF-7/TAM-shChk-α cells time-dependently. (B) Western blot analysis of the LC3. 3-MA led to a significant reduction of elevated LC3 in MCF-7/TAM-shChk-α cells after treatment with 3-MA. (B) Western blot analysis of the p62. 3-MA caused to a slight decrease in p62 level in MCF-7/TAM-shChk-α cells. Data are presented as the mean ± standard deviation of 5 independent experiments. *p<0.05. 3-MA vs untreated.

The extent of autophagy induction by shChk-α is same in case of both MCF-7 cells and MCF-7/TAM cells. We explored what happens to MCF-7/TAM and MCF-7/TAM-shChk-α cells in terms to viability and death like if autophagy is being inhibited. After treatment with 3-Methyladenine (3-MA), an autophagy inhibitor, cell viability and LC3 and p62 expression in MCF-7/TAM and MCF-7/TAM-shChk-α cells were assessed by MTT assay and Western blot. The treatment with 3-MA (5 mM) caused to cell death time-dependently (Fig 6A). At 24h 3-MA decreased the cell viability up to 86.8±1.7% and 87.3±2.6% in MCF-7/TAM and MCF-7/TAM-shChk-α relative to untreated cells. At 48h the cell viability of MCF-7/TAM and MCF-7/TAM-shChk-α was 69.8±1.1% and 78.2±2.6%, respectively (*p<0.05). At 72h 3-MA reduced the cell viability up to 50.6±4.4% and 52.0±2.3% in MCF-7/TAM and MCF-7/TAM-shChk-α (*p<0.05). 3-MA at the concentration that induced cell death attenuated the increased levels of LC3 but not p62 in MCF-7/TAM and MCF-7/TAM-shChk-α (Fig 6B). From these results, the autophagy is being in MCF-7/TAM and MCF-7/TAM-shChk-α cells pays a role in protecting the cells from TAM.

## Discussion

Currently, endocrine therapy using TAM has led to a significant improvement in outcomes for patients with ER+ breast cancer. However, in contrast to ER- f breast cancer, ER+ breast cancer has an unfortunate propensity to recur after decades [2]. In the case of late recurrence, cells that acquire therapeutic resistance undergo autophagy, survive in a dormant state and give rise to incurable metastases [1,2]. We investigated the relationship between Chk-α and autophagy in TAM-resistant BCCs. The result demonstrates that autophagy induced Chk-α downregulation is responsible for cell dormancy or death control depending on the resistance and response to TAM. [14]. In our study, the high expression of Chk-α and elevated levels of the choline-metabolites PC observed in TAM-resistant BCCs. These data provided evidence that high expression and activity of Chk-α along with high level of PC levels can play a role in mediating TAM resistance and Chk-α downregulation may provide an alternative to abrogate the TAM resistance and enhance the therapeutic response to anti-esteogen, TAM in ER+ cancer cells and tumors. Autophagy is dysregulated in several disorders, including cancer and has become an attractive target for anti-cancer therapies [20]. Autophagy is an adaptive response that enables cancer cells to survive in the injuries caused by treatments such as chemotherapy and radiation therapy [28]. Inhibition of autophagy constitutes a novel therapeutic mechanism for treatment of resistant tumors. Autophagy assessed by the accumulation of autophagosomes is frequently upregulated in breast cancer cells following treatment with TAM or imatinib [22,23]. Consistent with other group [20,23], we found that the expression of cleaved LC3 leading to the induction of autophagosome formation as a marker for autophagy was increased in TAM-MCF-7 cells. The role of autophagy in cancer remains ambiguous, and mechanisms that induce autophagy and regulate its outcome in human cancers are poorly understood. Several oncogenes (PI3K, AKT and Bcl-2 family) and tumor suppressors (beclin-1, tumor protein p53 (TP53), death-associated protein kinase-1 (DAK-1), phosphatase and tensin homolog (PTEN), aplasia Ras homolog member I (ARHI) contribute to the autophagic pathways, and loss of their function could promote or inhibit the induction of autophagy [29–31]. We first found that downreguation of expression and activity of Chk-α enhanced LC3 expression in BCCs, thereby leading to autophagy. Based on these data, we can suggest the existence of a crosstalk between choline metabolism and autophagy, eventually leading to either cell survival or cell death depending on drug sensitivity and resistance. Targeting autophagy in BCCs could be a potential strategy for drug development to overcome TAM resistance of ER+ breast cancers.

There are complex interactions of oncogenic signaling in therapeutic resistance, choline metabolism and autophagy. Deregulation of the PI3K/AKT and mTOR pathways has been shown to be involved in doxorubicin- and TAM-resistance in breast cancer through the regulation of multidrug resistance proteins [32,33]. The MAPK pathway influences chemotherapeutic drug resistance as ectopic activation to doxorubicin and paclitaxel in BCCs [34,35]. The block of the PI3K/AKT signaling pathway inhibits choline uptake by the regulation of choline transport, and PI3K/AKT signaling is also associated with Chk-α activation [36]. The downregulation of Chk-α simultaneously attenuates PI3K/AKT and MAPK signaling, inhibits cell proliferation and markedly decreases anchorage-independent survival [37]. Treatment with PI-103, a PI3K and mTOR inhibitors resulted in a concentration- and time-dependent decrease in PC and Chk-α levels in human prostate and colon carcinoma cell lines [38]. The aforementioned reports and our study propose the strong positive correlation between Chk-α and oncogenic signaling pathway. In turn, the decreased Chk-α further may attenuate the MAPK, PI3K and mTOR signaling pathway through a positive feedback loop. Furthermore, the activation of the PI3K/AKT and mTOR signaling pathways promotes necrotic cell death via the suppression of autophagy [24]. Autophagy-related LC3 is increased in cells treated with a PI3K/AKT or mTOR inhibitor [39,40]. These findings are consistent with our results that the phosphorylations of AKT, mTOR and EKR1/2 were interrupted in Chk-α-downregulated BCCs, which is associated with induction of autophagy. Cancer dormancy observed in the local recurrences or metastasis is a relevant problem in clinic because the dormant tumor cells have drug resistance mechanisms [3]. Targeting certain enzymes such as Chk-α, which are related to choline metabolism, provides promising therapeutic opportunities for tumor growth arrest [9,11,41]. Cyclin-dependent kinase inhibitors, p21 and p27 act as a tumor suppressor for promoting anti-proliferative activities and typically cause cells to arrest in the G0/G1 phase of the cell cycle, which confer to dormancy [42]. Ki67 expressed by proliferating cells in all phases of the active cell cycle (G1, S, G2 and M) is currently the proliferation biomarker [43]. We here found that the majority of TAM-resistant BCCs existed in G0/G1 phase and Chk-α downregulation led to a significant enhancement in G0/G1 population. Meanwhile Ki67-positive cells were higher in the parent cells MCF-7 as compared to TAM-resistant cells, MCF-7/TAM and Chk-α downregulation caused BCCs to the decreased Ki67-positive cells. Autophagy appears to play an important role in the mechanism of keeping cell dormancy and survival [20,29]. Certain cancer forms, such as pancreatic cancer, multiple myeloma and triple-negative breast cancer, have also been demonstrated to be dependent on autophagy for their growth [44–46].

Wu *et*. *al*. reported that 3-MA, which is well known as an autophagy inhibitor had dual role in modulation of autophagy via different temporal patterns of inhibition on class I and III PI3K [47]. Thus, 3-MA was found to promote autophagy flux and increase the autophagic markers, LC3 by blocking class I PI3K persistently when treated under nutrient-rich culture medium with a prolonged period of treatment, whereas it is capable of suppressing starvation-induced autophagy by blocking class III PI3K transiently. Our initial intention was to examine the inhibitory effects of 3-MA on the expression of LC3 increased by downregulation of Chk-α under nutrient-rich conditions. We observed that 3-MA treatment did not result in visible decrease in LC3 in control cells and led to a decrease in the elevated LC3 expression in shChk-α cells. However, in our experimental condition, an incubation with autophagy inhibitor 3-MA did not result in visible and large decrease in LC3 expression in both cells. These results may due to different dual role related with the promotion of autophagy flux and the suppression of maturation of autophagosomes. We here found treatment 3-MA induced the cell death in MCF-7/TAM and MCF-7/TAM-Chk-α cells. These results provided that autophagy enhanced by Chk-α downregulation might be play a role in protecting TAM-resistant BCCs from a hormone therapy drug.

In conclusions, Together with our findings regarding the autophagy regulated by Chk-α expression and activity, we suggest the existence of the link between choline metabolism, Chk-α and PC and autophagy. The downregulation of Chk-α expression and activity induced the autophagy through upregulating LC3 expression in BCCs and interrupted PI3K/AKT, mTOR and MAPK signaling pathway, resulting in induction of autophagy. ER+ BCCs exhibited the induction of the autophagy as well as caspase-dependent cell death by the downregulation of Chk-α. Meanwhile, autophagy is being in TAM-resistant BCCs, MCF-7/TAM and MCF-7/TAM-shChk-α contributed to inhibit the death. The downregulation of Chk-α in TAM-resistant BCCs led to the further enhanced autophagy and G0/G1 phase fraction and attenuated cell growth rate, triggering cancer dormancy. Finally, our study will be useful to understand the interplay between choline metabolism and autophagy and explore new therapeutic targets and develop novel treatment strategies that preferentially target TAM-resistant BCCs.

## Supporting Information

S1 Fig Choline kinase-α (Chk-α) and LC3 I/II expression increased in MCF-7 cells treated with tamoxifen. (A) RT-PCR analysis of Chk-α in MCF-7 treated with tamoxifen for 24 h. (B) Western blot analysis of LC3 I/II in MCF-7 cells treated with tamoxifen for 24 h.
(TIF)

S2 Fig Beclin-1 expression was not altered in Chk-α-downregulated breast cancer cells. Western blot analysis of beclin-1 in control (MCF-7 and MCF-7/TAM) and shChk-α-transduced (MCF-7-shChk-α and MCF-7/TAM-shChk-α) cells.
(TIF)
