# Supplementary figures and images for: Downregulation of Choline Kinase-Alpha Enhances Autophagy in Tamoxifen-Resistant Breast Cancer Cells
Source: PLoS One. 2015 Oct 23;10(10):e0141110. doi: 10.1371/journal.pone.0141110 (PMC4619766; doi:10.1371/journal.pone.0141110)

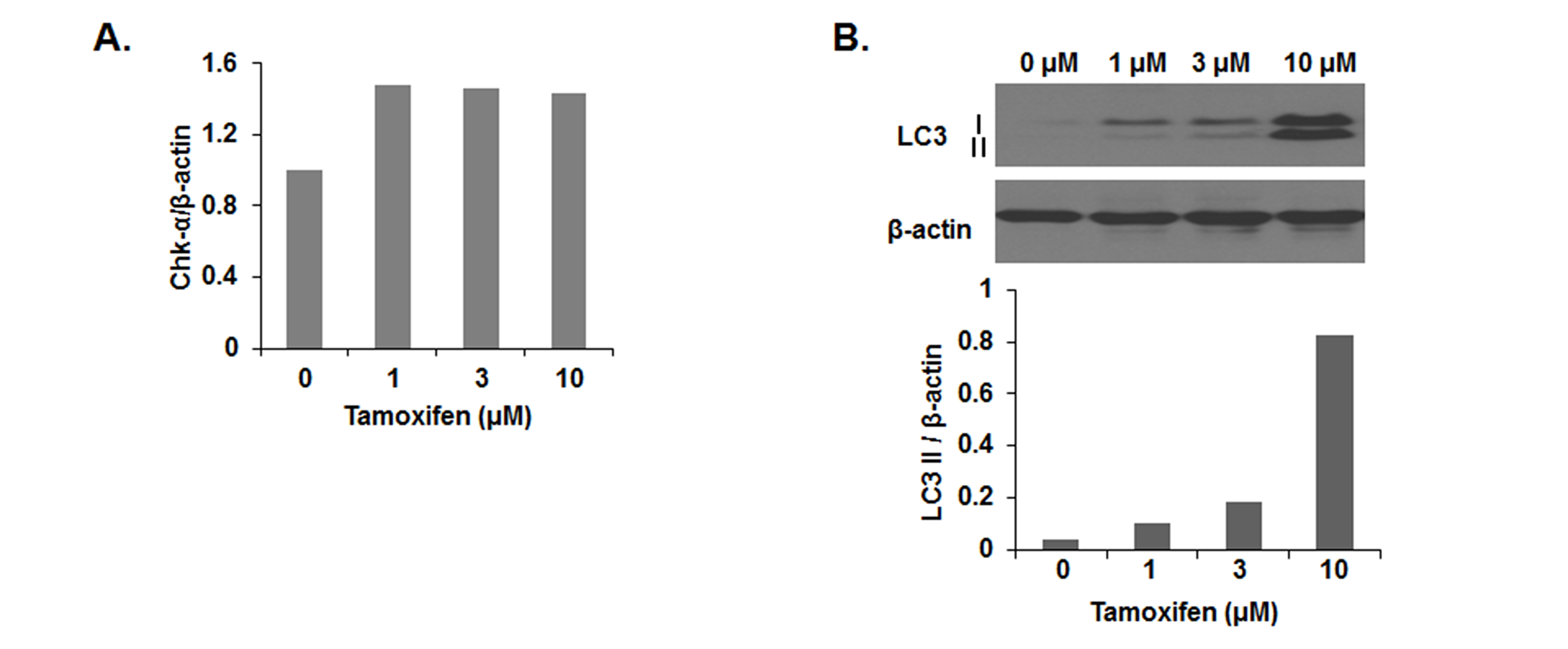

Supplement: S1 Fig — (A) RT-PCR analysis of Chk-α in MCF-7 treated with tamoxifen for 24 h. (B) Western blot analysis of LC3 I/II in MCF-7 cells treated with tamoxifen for 24 h. (TIF) [file pone.0141110.s001.tif]

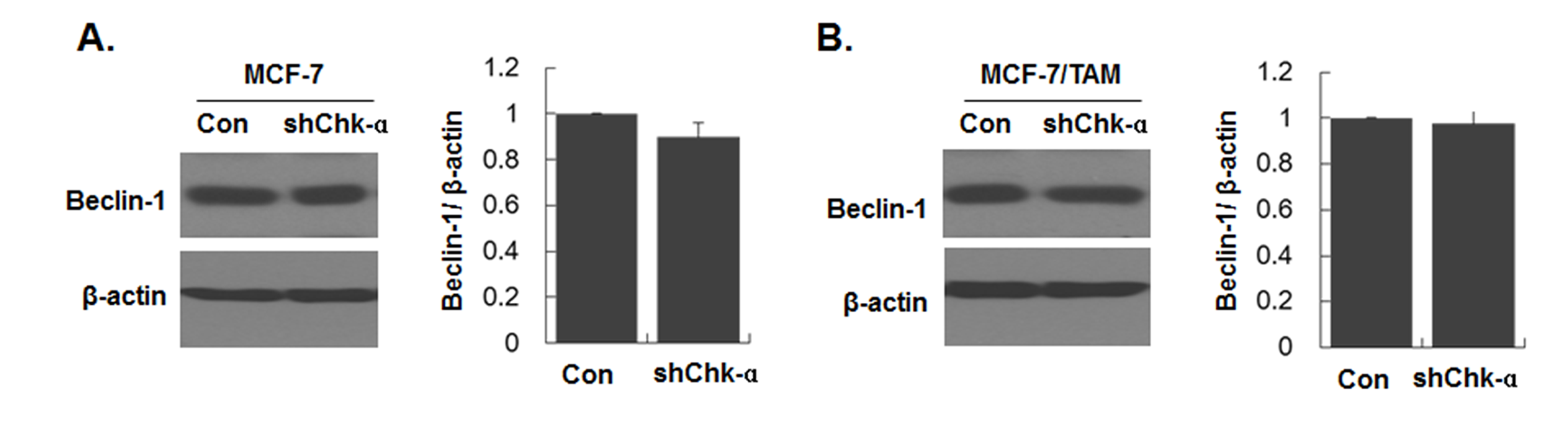

Supplement: S2 Fig — Western blot analysis of beclin-1 in control (MCF-7 and MCF-7/TAM) and shChk-α-transduced (MCF-7-shChk-α and MCF-7/TAM-shChk-α) cells. (TIF) [file pone.0141110.s002.tif]
